# Incorporating thresholds into understanding salinity tolerance: A study using salt‐tolerant plants in salt marshes

**DOI:** 10.1002/ece3.3209

**Published:** 2017-07-05

**Authors:** Qiang He, Brian R. Silliman, Baoshan Cui

**Affiliations:** ^1^ School of Environment State Key Laboratory of Water Environment Simulation Beijing Normal University Beijing China; ^2^ Division of Marine Science and Conservation Nicholas School of the Environment Duke University Beaufort NC USA

**Keywords:** coastal wetlands, nonlinear ecological processes, *Salicornia europaea*, salt marsh, soil salinity, *Suaeda salsa*

## Abstract

Although salinity in many ecosystems such as salt marshes can be extremely high, an asymmetry in salinity range between experimental studies (relatively narrow) and field conditions (potentially broad) has strongly affected current understanding of plant salinity tolerance. To improve understanding, it is thus important to examine plant tolerances over a broad range of salinities and identify potential tolerance thresholds. We examine tolerances of two widely distributed marsh plants, *Suaeda salsa* and *Salicornia europaea*, to salinities ranging from 0 to 100 g/kg, and determine survival, above‐ and belowground biomass after 8 weeks of salinity treatment. Both species, *Sa. europaea* in particular, have much broader salinity tolerances than other plants previously examined, (2) plant survival, above‐ and belowground biomass have remarkably different responses to salinity, and (3) there is a nonlinear, threshold response of *S. salsa* to salinity, above which *S. salsa* survivorship drastically decreases. These results provide multiple important insights. Our study suggests that the potential for using these halophytes to revegetate and restore salt‐affected land may be greater than previously thought, and highlights the importance of studying multiple plant responses. Importantly, our study calls for a better integration of thresholds into understanding plant salinity tolerances and their applications.

## 1 INTRODUCTION

Salinization is a detrimental environmental problem that threatens many natural and artificial ecosystems worldwide. Salinization associated with groundwater and irrigation affects ~16% of the world's agricultural ecosystems (Rengasamy, 2006); increases in roadways and deicer use have been shown to salinize freshwaters across Europe and the United States (Kaushal et al., 2005; Löfgren, 2001); climate change, drought, decreases in estuarine freshwater flow, sea‐level rise, and storm surges have been suggested to increase the extent and severity of salinization in coastal wetlands globally (Herbert et al., 2015). Under this trend in salinization worldwide, managing and predicting the impact of salinization on ecosystems require a better understanding of salinity tolerance in plants and other primary producers that are often the foundations of ecosystems.

Plant salinity tolerance has been studied intensively for a long time. It is well known that plants widely differ in salinity tolerance, and those that tolerate salt concentrations (~200 mmol/L NaCl or 11.5 g/kg) that kill 99.8% of other species are often called halophytes (definitions of halophytes can vary; Flowers & Colmer, 2015). Halophytes are equipped with a number of strategies to tolerate salinity, including at least selective accumulation or exclusion of ions, control of ion uptake and transport, and compartmentalization of ions (Parida & Das, 2005). The effects of increasing salinity on plant performance, however, vary remarkably even within halophytes. Many halophytes generally grow optimally in freshwater (Flowers & Colmer, 2008; Munns & Tester, 2008), while some could show optimal growth in low salinities. In either case, it has been implicated that most halophytes die or show strongly reduced growth in salinities around that of seawater (Flowers, Galal, & Bromham, 2010; Greenway & Munns, 1980).

Salinities in many ecosystems, however, can be substantially higher than seawater salinity. In salt marshes, for example, soil pore water salinity can be several times that of seawater (70–150 g/kg), especially in regions with a dry climate (e.g., California, southeastern United States, and temperate China) or in upper marsh zones where salt concentration by evaporation is intense (Cui, He, & An, 2011; Hoffman & Dawes, 1997; Hsieh, 2004; Pennings & Bertness, 2001). Even in New England, salt marshes with a rainy, cold climate, soil salinity can be up to 50–60 g/kg (Bertness, Gough, & Shumway, 1992; Shumway & Bertness, 1992). Although hypersalinity stress limits relatively salt‐sensitive marsh grasses, some halophytes such as *Salicornia* spp. could colonize hypersaline marsh areas (Bertness et al., 1992; Pennings & Callaway, 1992; Shumway & Bertness, 1992), indicating their great capacity to tolerate hypersalinity stress.

Despite the potential for plants to tolerate such high levels of salinity stress, the majority of past studies, even those from salt marshes, examined relatively small gradients of salinity, typically up to ~40 g/kg (Bertness et al., 1992; Egan & Ungar, 2001; Howard & Rafferty, 2006; Huckle, Potter, & Marrs, 2000; Katschnig, Broekman, & Rozema, 2013; Kuhn & Zedler, 1997; Phleger, 1971; Redondo‐Gómez et al., 2007; Ungar, Benner, & McGraw, 1979). Much fewer studies examined plant tolerance to salinities as high as 50–60 g/kg (Guo & Pennings, 2012; He, Cui, & An, 2011; He, Cui, Bertness, & An, 2012; Khan, Ungar, & Showalter, 2000; Yeo & Flowers, 1980). However, in these studies, highly salt‐tolerant plants often survived and continued biomass accumulation (although at lower rates), and their salinity tolerance thresholds are unknown. A few studies that examined broader salinity gradients up to 70–100 g/kg suggested, without specific statistical tests, that salinity tolerance thresholds might exist (Crain, Silliman, Bertness, & Bertness, 2004; Li, Liu, Khan, & Yamaguchi, 2005). Such studies, however, are rare and have often focused on a single plant response variable (e.g., aboveground biomass). Other plant response variables, especially plant survival, can greatly differ in response to environmental stress (He, Bertness, & Altieri, 2013). Clearly, there is a significant asymmetry in salinity range between experimental studies and documented field conditions in the literature, and our current understanding of plant salinity tolerance has been strongly affected and limited due to this asymmetry. To improve current understanding, it is thus important to examine plant tolerances over a broad range of salinities and to identify potential tolerance thresholds above which salinity stress will lead to a drastic decline in plant survival/growth.

**Figure 1:**
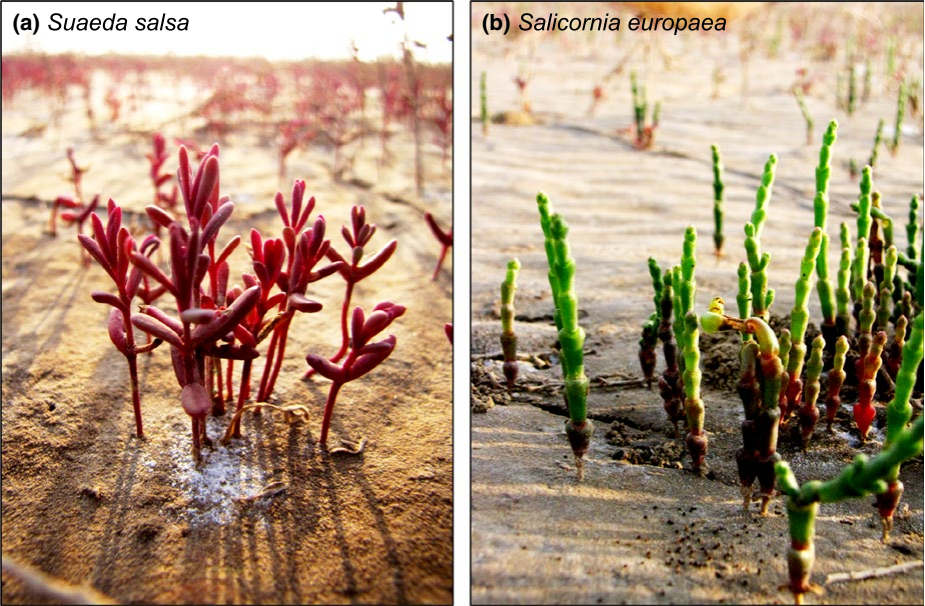
Photographs showing *Suaeda salsa* (a) and *Salicorina eutropaea* (b) in a hypersaline salt marsh in the Yellow River Delta, northern China. Photo credits: QEcology.org

Here, we examined salinity tolerances of two obligate halophytes, *Suaeda salsa* (Linnaeus) Pallas and *Salicornia europaea* L. (Fig. 1), species widely distributed in salt marshes and saline drylands. *Suaeda salsa* has been found across northeast Asia, and *Sa. europaea* across Asia, Europe, North America, and Africa (He, Altieri, & Cui, 2015; Muscolo, Panuccio, & Piernik, 2014). Both *S. salsa* and *Sa. europaea* are in the family of Amaranthaceae and are among the salt‐tolerant plants known in salt marshes (He et al., 2015). Although salinity tolerances of *S. salsa* and *Sa. europaea* have been examined in a number of studies, as discussed above, past studies often examined a relatively small range of salinities, despite the fact that these species can potentially survive much higher salinities. Furthermore, we know of no study that has determined potential thresholds in the salinity tolerance of these species along a broad range of salinity and compared their salinity tolerance thresholds. Identifying potential salinity tolerance thresholds is crucial, as a relatively small change around this point can lead to a drastic decline in plant performance and ecosystem productivity (Brook, Ellis, Perring, Mackay, & Blomqvist, 2013).

We investigated the responses of *S. salsa* and *Sa. europaea* survival and growth to a broad range of soil pore water salinities (0–100 g/kg) in a pot experiment. We aimed to examine whether these plants can tolerate salinity stresses times that of seawater and whether there are thresholds in their salinity tolerance. Specifically, we hypothesized that: (1) both species perform optimally in low‐salinity treatments and could well survive much higher salinity stress than that of seawater; (2) *Sa. europaea* has a greater capacity to tolerate salinity stress than does *S. salsa*; and (3) there are critical thresholds in the salinity tolerances of these two species.

## 2 MATERIALS AND METHODS

Experimental work was conducted at our field station (37°50″N, 119°02″E) in the Yellow River Delta, northern coastal China. The Yellow River Delta has a warm temperate climate, with dry falls, winters and springs, and rainy summers. The long‐term average annual precipitation is 537.3 mm, and the long‐term average annual temperature of 12.8°C (see He et al., 2015 and references therein). Salt marshes in the Yellow River Delta are primarily dominated by *S. salsa*. *Salicornia eutropaea* occurs often as a subordinate in *S. salsa* communities (He et al., 2015). Other plant species that exist in the Yellow River Delta include *Phragmites australis*,* Tamarix chinensis*, and the invasive cordgrass *Spartina alterniflora* (Cui et al., 2011). Salt marshes are flooded irregularly by semidiurnal microtides. The marsh platform is often hypersaline, and soil salinities can vary greatly from 30 to 200 g/kg, with apparent salt accumulation on the soil surface, especially in dry, low‐rainfall periods (Cui et al., 2011; He et al., 2015).

The experiment had 11 levels of salinity treatment (0–100 g/kg, with 10 g/kg intervals) replicated six times for each of the two study species *Sa. europaea* and *S. salsa* (*n *= 132 pots in total). In early June 2013, we excavated using a soil corer 100 soil blocks (7.5 cm in diameter, 10 cm in depth) each containing >30 *Sa. europaea* and *S. salsa* seedlings, respectively, from a high marsh area in the Yellow River Estuary (i.e., intertidal populations; Song, Shi, Gao, Fan, & Wang, 2011). Each of these soil blocks was transplanted into a plastic pot of 2.5 L. All pots were placed in a common garden under natural light and temperature conditions, except that precipitation was excluded by a rain shelter made of transparent plastic. After a two‐week acclimatization period, during which we watered the plants with freshwater, we thinned the plants in each pot to 10 individuals of similar size (5–8 cm for *Sa. europaea*, and 7–10 cm for *S. salsa*) and assigned them to each of the 11 salinity treatments. The density of 10 individuals in each pot was within their natural range. To control for soil pore water salinity, we placed pots into a shallow layer (~4–6 cm) of standing water of different salinities and allowed the treatment solution to rise to the soil surface. This way, soil pore water was composed of the treatment solution, and soil pore water salinity would be consistent with that of the treatment solution. This is a common method to control soil pore water salinity levels in plant salt tolerance studies and has been considered to be effective and efficient (e.g., Cao et al., 2006; Crain et al., 2004; English & Colmer, 2011; He et al., 2012; Soriano et al., 2014; Youngman & Heckathorn, 1992). We monitored and adjusted salinities at least every other day by adding freshwater or sea salt. Salinities were increased gradually (by 10–20 g/kg every 2 days) to avoid shock, and all salinity treatments were in force after 10 days. Eight weeks later, the number of *Sa. europaea* and *S. salsa* survivors in each pot was counted, aboveground biomass harvested, oven‐dried at 60°C for 48 hr and weighed. Plant roots were rinsed in tap water, oven‐dried, and weighed to quantify belowground biomass.

We used one‐way ANOVAs followed by Tukey HSD multiple comparisons to test for the effects of different salinity treatments on population‐level (above‐ and belowground biomass per pot) and individual‐level (above‐ and belowground biomass per plant survivor) performances of *S. salsa* and *Sa. europaea*. Data of *S. salsa* belowground biomass per pot were square root transformed to meet the normality assumption of ANOVA. *Suaeda salsa* and *Sa. europaea* survivorship data did not meet the normality assumption of ANOVA even after normal transformations, so Kruskal–Wallis tests and nonparametric multiple comparisons (Dunn All Pairs for Joint Ranking) were used instead. ANOVAs and Kruskal–Wallis tests were conducted using JMP 10 (SAS Institute, Cary, NC). Optimal and threshold salinity levels were determined using the *breakpoints* function (minimal segment size was set to be 13; so each segment would have at least data from three salinity levels) in the *strucchange* package in R 3.1.2 (R Core Team, 2015). We preferred to use the *breakpoints* method instead of the two‐piece, threshold–slope response function or the compound–discount function (Steppuhn, Van Genuchten, & Grieve, 2005) to determine optimal and threshold salinity levels, as the response curves of the halophytes we examined here did not always follow that of agricultural crops.

## 3 RESULTS

**Figure 2:**
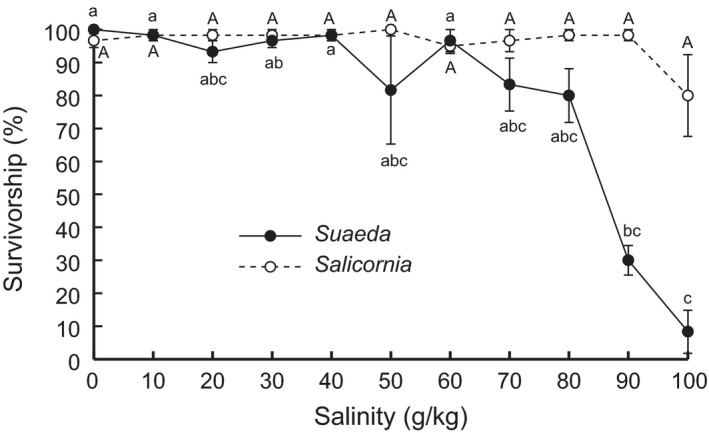
Effects of 0–100 g/kg salinity treatments on the survivorship of *Suaeda salsa* and *Salicornia europaea*. Data are means ± *SE* (*n *= 6). Within each species, data points sharing a letter are not significantly different from one another (*p *> .05)

At the population level, salinity treatments of <90 g/kg had significant effects on the survival of neither *S. salsa* nor *Sa. europaea* (*p *> .05; Table 1, Fig. 2). However, the effect of increasing salinities on *S. salsa* survival had two break points—50 and 80 g/kg, respectively (Table 2): *S. salsa* survival slightly decreased with salinity treatments of >60 g/kg and showed sharp declines when salinities increased to 90 and 100 g/kg, where ~30 and 10% of *S. salsa* plants survived, respectively (Fig. 2). By contrast, *Sa. europaea* survival was not significantly reduced even in 100 g/kg salinity treatments (Fig. 2), and no break point in the effect of increasing salinities on *Sa. europaea* survival was found (Table 2).

**Table 1:** Summary of test statistics for the effects of salinity treatments on different performance measures of *Suaeda salsa* and *Salicornia europaea*. All tests were one‐way ANOVAs, except that Kruskal–Wallis tests were used for survivorship of both *S. salsa* and *Sa. europaea* and for *S. salsa* belowground biomass per pot

Response variable	*Suaeda salsa*			*Salicornia europaea*		
	*df*	*F* (χ^2^)	*p*	*df*	*F* (χ^2^)	*p*
Survivorship	10	42.99	<.0001	10	12.44	.26
Aboveground biomass per pot	10, 55	14.07	<.0001	10, 55	9.81	<.0001
Belowground biomass per pot	10	51.65	<.0001	10, 55	9.83	<.0001
Aboveground biomass per survivor	10, 50	5.77	<.0001	10, 55	10.2	<.0001
Belowground biomass per survivor	10, 50	8.57	<.0001	10, 55	10.23	<.0001

**Table 2:** Break points (salinity levels in g/kg) in the effect of increasing salinities on the survivorship and growth of *Suaeda salsa* and *Salicornia europaea*. NA indicates no break points

Response variable	*Suaeda salsa*	*Salicornia europaea*
Survivorship	50; 80	NA
Aboveground biomass per pot	20; 50; 80	30
Belowground biomass per pot	NA	30
Aboveground biomass per survivor	NA	30
Belowground biomass per survivor	70	30

**Figure 3:**
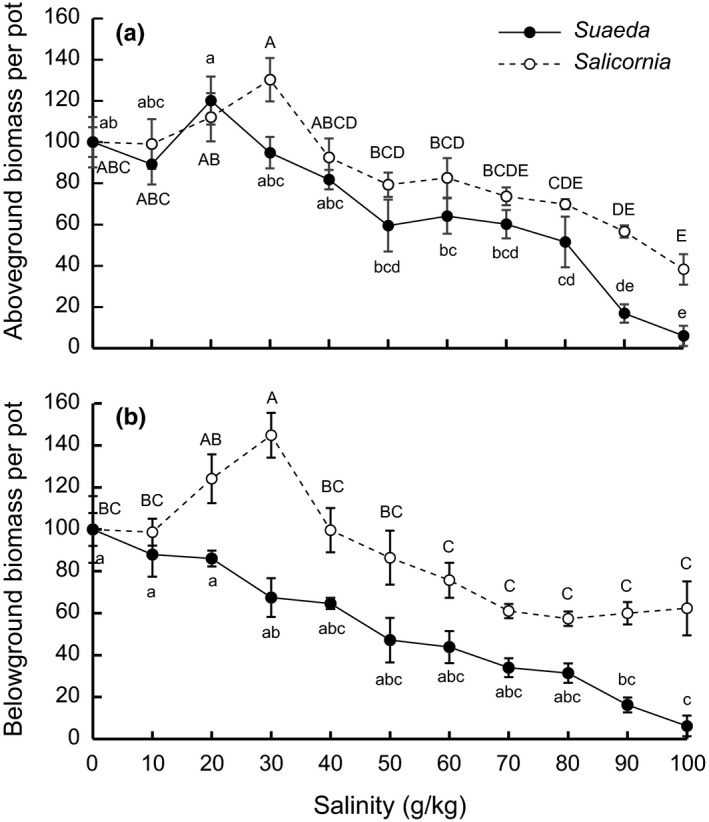
Effects of 0–100 g/kg salinity treatments on the aboveground (a) and belowground (b) biomass of *Suaeda salsa* and *Salicornia europaea* per pot. Data are means ± *SE* (*n *= 6). To facilitate comparison between species, biomass data are shown as percentages of plant performance in 0 g/kg salinity treatments. Within each species, data points sharing a letter are not significantly different from one another (*p *> .05)

For both *Sa. europaea* and *S. salsa*, aboveground biomass reached a peak (130% and 120% of that in 0 g/kg salinity treatments, respectively) at low‐salinity treatments (Table 1, Fig. 3a), although the optimal salinity was higher (30 g/kg) for *Sa. europaea* than that for *S. salsa* (20 g/kg). Further higher salinities generally decreased aboveground biomass of both *Sa. europaea* and *S. salsa* (Fig. 3a). However, there were two break points for *S. salsa*—50 and 80 g/kg, respectively (Table 2). Increasing salinities between 20 and 50 g/kg and between 80 and 100 g/kg led to rapid declines in *S. salsa* aboveground biomass, while differences in *S. salsa* aboveground biomass between salinity treatments of 50 and 80 g/kg were minor (Fig. 3a). *Suaeda salsa* belowground biomass was gradually reduced by increasing salinity, and there were no optimal or threshold responses. By contrast, *Sa. europaea* belowground biomass increased up to 145% of that in 0 g/kg salinity treatments by increasing salinities up to 30 g/kg, and generally decreased with further higher salinities (Fig. 3b).

**Figure 4:**
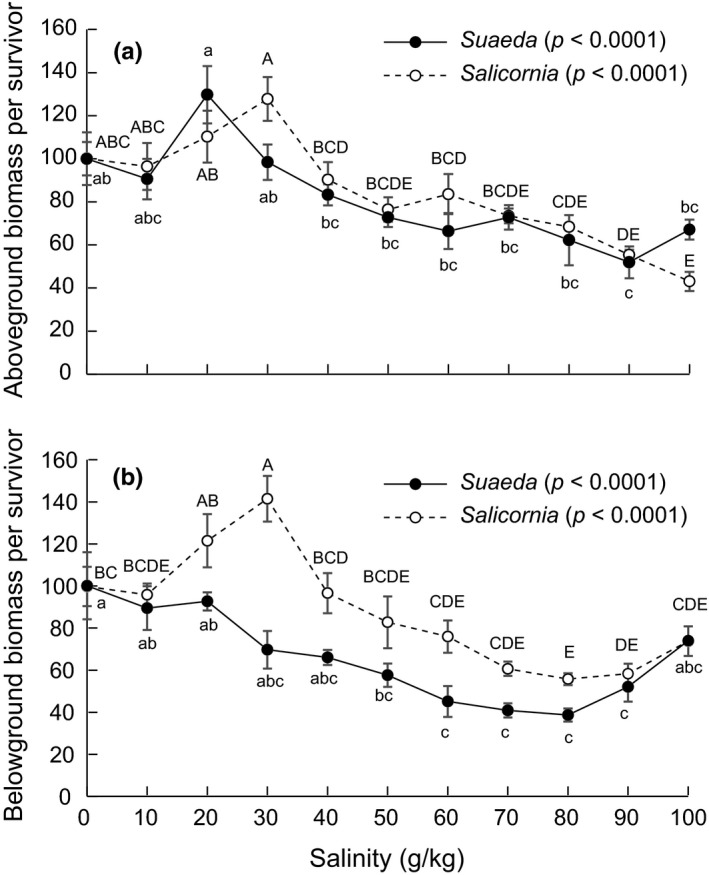
Effects of 0–100 g/kg salinity treatments on the aboveground (a), and belowground (b) biomass of *Suaeda salsa* and *Salicornia europaea* per survivor. Data are means ± *SE*. To facilitate comparison between species, biomass data are shown as percentages of plant performance in 0 g/kg salinity treatments. Within each species, data points sharing a letter are not significantly different from one another (*p *> .05)

When data were analyzed at the individual level for plant survivors, we found generally similar effects of salinity treatments (Fig. 4). However, high‐salinity treatments between 50 and 100 g/kg had generally minor effects on above‐ and belowground biomass of *Sa. europaea* and *S. salsa* plants that survived these salinity treatments, with above‐ and belowground biomass as high as 50%–80% of that in 0 g/kg salinity treatments. No break point for *S. salsa* aboveground biomass was detected (Table 2), although a break point of 70 g/kg was found for belowground biomass (Table 2): *S. salsa* belowground biomass generally decreased with increasing salinity before this salinity level, but slightly increased after this salinity level (Fig. 4b).

## 4 DISCUSSION

These results support our hypothesis that both *S. salsa* and *S. europaea* perform optimally in low‐salinity treatments (20–30 g/kg) and can survive much higher salinity stresses than the highest salinity levels investigated in the majority of past plant salinity tolerance studies (including studies on salt marsh plants). Our results also show that *Sa. europaea* has a greater capacity to tolerate salinity stress than does *S. salsa*:* Sa. europaea* can well survive salinities even exceeding 100 g/kg, while *S. salsa* has a salinity tolerance threshold of ~80 g/kg, above which higher salinities lead to a sharp decline in survival and growth. Our findings from a hypersaline ecosystem have multiple important implications for understanding plant salinity tolerances and their applications.

### 4.1 Salinity tolerances of *S. salsa* and *Sa. europaea*

Our results demonstrate the broad salinity tolerance of *S. salsa* and *Sa. europaea*, showing that both species can survive much higher levels of salinity stress than previously reported. Past studies suggested that *Sa. europaea* grow best at ~5–17.5 g/kg salinity treatments (85–300 mmol/L NaCl) and then decrease with increasing salinity (Moghaieb, Saneoka, & Fujita, 2004; Aghaleh, Niknam, Ebrahimzadeh, & Razavi, 2009; Fan et al., 2011; see Katschnig et al., 2013 for a recent review on the salt tolerance of *Salicornia* spp.). In our study, however, the optimal salinity for *Sa. europaea* was higher at 30 g/kg. In another study (Crain et al., 2004), although no optimal salinity was found, *Sa. europaea* biomass was consistently high among salinity treatments ranging from 0 to 70 g/kg. Egan and Ungar (2001) found that *Sa. europaea* biomass did not differ among 5, 10, and 20 g/kg salinity treatments. Gul, Ansari, and Khan (2009) reported that another species in the same genus, *Salicornia utahensis*, grew best at ~35 g/kg salinity treatments in a Pakistan salt desert. Differences in species adaptation, local climate, and experimental methodology may contribute to variation in the reported optimal salinity. Studies focusing on plant physiological responses (e.g., Aghaleh et al., 2009; Fan et al., 2011; Moghaieb et al., 2004) often used NaCl and sterilized sand for salinity treatments and grew plants in climate chambers, while ecological studies such as ours and Crain et al. (2004) often used sea salt and field‐collected soils and grew plants in a glasshouse or outdoor common garden. Although how salinity treatments were enforced and maintained were generally similar between our study and Crain et al. (2004), multiple plant individuals per pot were used in our study in contrast to one per pot as used in Crain et al. (2004), and our study may thus have allowed individual‐level variation in plant performance within a salinity level to be reduced and therefore average plant performance better represented.

*Suaeda salsa* has been less studied than *Sa. europaea*, but multiple past studies also reported that *S. salsa* performed best in low‐salinity treatments (~3–12 g/kg) rather than in 0 g/kg salinity treatments and then decreased by ~40%–50% with increasing salinity up to 35 g/kg (Duan, Li, Liu, Ouyang, & An, 2007; Song et al., 2009, 2011; see Song & Wang, 2015 for a recent review on the salt tolerance of *S. salsa*), in broad agreement with our results. However, the optimal salinity was found to be higher in our study. As discussed above, plant physiological studies such as Song et al. (2011) and Duan et al. (2007) often used NaCl and sterilized sand for salinity treatments and grew plants in climate chambers, while ecological studies often used sea salt and field‐collected soils and grew plants in glasshouses or outdoor common gardens. The former has the advantage of fully controlled indoor experimental settings, while the latter has the advantage of better simulating natural conditions. Indeed, our past experiments under similar conditions (sea salt and field‐collected soil substrates), but with a limited range or number of salinity levels (He et al., 2012, 2015), found no difference in *S. salsa* growth among salinity treatments ranging from 20 to 60 g/kg, although 90 g/kg salinity treatments substantially reduced *S. salsa* growth by 70%–90%, generally supporting the broad salinity tolerance we found in this experiment.

### 4.2 Importance of studying multiple plant responses

Our study highlights the importance of studying multiple plant responses that include survival and belowground biomass in understanding plant salinity tolerance. Many past studies on salinity tolerance often examined plant growth using aboveground biomass. Our results, however, suggest that the effects of salinity on plant survival can remarkably differ from those on plant aboveground biomass. *Suaeda salsa* and *Sa. europaea* plants that survived high salinities were able to maintain a relatively high biomass. At high salinities, population‐level biomass decline in *S. salsa* was primarily driven by plant mortality, not by a reduction in the biomass of surviving plants. Furthermore, although stimulation of aboveground biomass by low salinities was found for both species, stimulation of belowground biomass was found only for *Sa. europaea*, not for *S. salsa*. Stimulation of belowground growth by low salinities has been previously reported for other salt marsh plants (Venables & Wilkins, 1978). In fact, the effects of salinity on plant belowground growth can vary as greatly as those on aboveground growth (Adam, 1993). Our finding of no salinity stimulation of *S. salsa* belowground biomass agrees with past studies (Song et al., 2009; Yang, Song, & Wang, 2010), although contrary results exist (Duan et al., 2007; Liu, Duan, Li, Tadano, & Khan, 2008; Song et al., 2009). Even in these studies, nevertheless, growth stimulation by salinity was often weaker for belowground than for aboveground. In contrast to *S. salsa*, stimulation of *Sa. europaea* belowground growth by low salinities was more consistent among studies (Cooper, 1982; Keiffer, McCarthy, & Ungar, 1994; Ungar et al., 1979). Differences in root tolerance to salinity may be a cause of the lower salinity tolerance of *S. salsa* survival relative to that of *Sa. europaea*.

Our study focused on the effects of salinity on plant survival, above‐ and belowground growth, as the effects of salinity stress on seed germination of *S. salsa* and *Sa. europaea* have been well studied. It has been known that seed germination may require salinities to be lower. Indeed, while we found both *S. salsa* and *Sa. europaea* grow optimally at low‐salinity conditions, seed germination of both species is often highest in freshwater rather than in low‐salinity treatments, and generally decreases with increasing salinity (Duan et al., 2007; Ungar, 1977). For *Sa. europaea*, it has been found that no seeds could germinate in the 5.0% NaCl solutions (~50 g/kg) (Ungar, 1977). For *S. salsa*, Duan et al. (2007) found that only ~6.3% of seeds germinated under 35 g/kg salinity treatments in comparison with 63.8% in 0 g/kg salinity treatments. However, much higher germination rates under high‐salinity treatments have been reported in other studies. Song et al. (2009) found that *S. salsa* seed germination remained ~71%–88% in 35 g/kg salinity treatments. Li et al. (2005) found that 10% of *S. salsa* brown seeds (*S. salsa* has dimorphic seeds—brown and black; see Wang et al., 2015; Song et al., 2016; Zhou et al., 2016) were still able to germinate in 70 g/kg salinities, although a sharp decline in germination rate occurred between 35 and 47 g/kg salinity treatments. In either case, high‐salinity stress did not affect the viability of *S. salsa* seeds, and nongerminated seeds in high‐salinity treatments germinated when salinity stress was lowered (Duan et al., 2007).

### 4.3 Incorporating thresholds into understanding plant salinity tolerance

Our results suggest that *Sa. europaea* and *S. salsa* have a much greater capacity to tolerate salinity stress than many other plants. Previous comparative studies also found *Sa. europaea* to be the most salt‐tolerant among nine marsh plants in southern New England (Crain et al., 2004) and 15 marsh plants in the Netherlands (Rozema, Luppes, & Broekman, 1985). He et al. (2012) compared the stress tolerances of *S. salsa* and *Suaeda glauca* and found *S. salsa* to have a much higher salinity tolerance. It is not surprising that salt marsh plants, including *Spartina alterniflora*,* Batis maritime*, and *Salicornia virginica,* can survive salinities as high as ~50 g/kg (Guo & Pennings, 2012). Our work on *Sa. europaea* and *S. salsa* shows that these plant species have even higher salinity tolerance limits than reported in past studies. The broad salinity tolerances of *Sa. europaea* and *S. salsa* have been attributed to their high Na^+^ and Cl^−^ accumulation capacities. Both species do not have salt glands or salt bladders, but have multiple sodium compartmentalization mechanisms and can accumulate considerable amounts of Na^+^ and Cl^−^ in their shoots (Lv et al., 2012; Ushakova, Kovaleva, Gribovskaya, Dolgushev, & Tikhomirova, 2005; Wang, Lüttge, & Ratajczak, 2001).

Importantly, although for both species there are often nonlinear relationships between performance and salinity stress, our study found that there was a critical tolerance threshold (80 g/kg) for *S. salsa* survival. The sharp decline in *S. salsa* survivorship under salinities of above 80 g/kg probably resulted from root functional failures (see above). Although our group has focused on the ecology rather than the physiology of these plants, we encourage that future studies investigate the physiological mechanisms underlying such thresholds. Similarly, Li et al. (2005) also found a drastic decline in the germination rate of *S. salsa* seeds (brown seeds) under salinities of above 35 g/kg. While we did not find a salinity threshold for *Sa. europaea*, Crain et al. (2004) found a drastic decline in the growth of *Sa. europaea* under salinities of above 70 g/kg. Thresholds in plant salinity tolerance have also been reported in other studies, often in crops (Maggio, Raimondi, Martino, & De Pascale, 2007; Steppuhn et al., 2005). In natural systems, for example, Koyro (2006) found a salinity threshold of 8.75 g/kg for *Plantago coronopus*, and Koch, Schopmeyer, Kyhn‐Hansen, Madden, and Peters (2007) found salinity thresholds of ~50–65 g/kg for three tropical seagrasses. Threshold responses to salinity and other stresses are likely common in many ecosystems. Given that anthropogenic factors including climate change, drought, and freshwater resource decline are increasing salinity stress in many ecosystems, guarding exceedance of salinity tolerance thresholds will be key to helping conserve these ecosystems.

## 5 CONCLUSIONS

Our study shows that (1) both *S. salsa* and *Sa. europaea*, especially *Sa. europaea*, have much broader salinity tolerances than other plants previously reported, (2) plant survival, above‐ and belowground biomass can have remarkably different responses to salinity, and (3) there is a nonlinear, threshold response of *S. salsa* to increasing salinity stress. Our findings highlight the great potential for these plants to endure salinity stress, and have important implications for understanding plant salinity tolerances and their applications. *Suaeda salsa* and *Sa. europaea* have been widely considered ideal for revegetation and remediation of salt‐affected land (Rozema & Schat, 2013; Song & Wang, 2015). Our work suggests that the potential for using these species to revegetate and restore salt‐affected land may be greater than previously thought. Our results also emphasize that such practices should incorporate salinity tolerance thresholds and avoid situations that exceed these thresholds via proper site selection, salinity reduction, and other measures, so success could be maximized. Understanding salinity tolerance thresholds in plants is an important first step to practical applications and predicting natural community dynamics that are also contingent on other factors (e.g., competition and herbivory; He et al., 2015) that affect plant performance.

## CONFLICT OF INTEREST

None declared.
